# Supplementary material for: Beyond PD-L1—Identification of Further Potential Therapeutic Targets in Oral Cancer
Source: Cancers (Basel). 2022 Apr 2;14(7):1812. doi: 10.3390/cancers14071812 (PMC8997752; doi:10.3390/cancers14071812)
Supplement: Supplementary file 1 [file cancers-14-01812-s001.zip › cancers-1638469-supplementary.pdf]

**Supplementary Tables and Figures**

Table S1. Correlation of the immune checkpoints PD1 and PD-L1/2 expression in tumor tissues. P value is calculated by Spearman's rho correlation. * The correlation is significant at the 0.01 level (2-tailed). ϱ= Correlation Coefficient. n= number of observations correlated.

| | correlation | PD1 | PD-L1 | PD-L2 |
|---|---|---|---|---|
| PD1 | ϱ<br>p value<br>n | 1.000<br><br>139 | | |
| PD-L1 | ϱ<br>p value<br>n | 0.093<br>0.276<br>139 | 1.000<br><br>139 | |
| PD-L2 | ϱ<br>p value<br>n | 0.05<br>0.558<br>139 | **0.257***<br>**0.002**<br>139 | 1.000<br><br>139 |

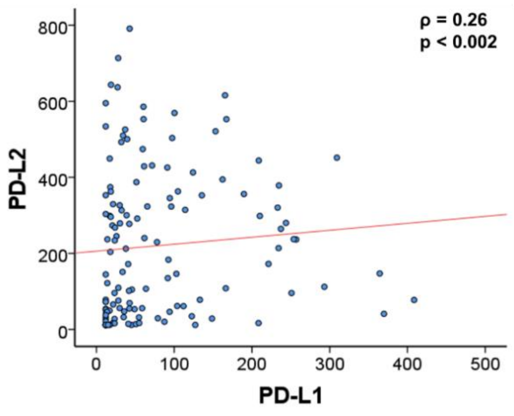


Figure S1. Moderate positive correlation between PD-L1 and PD-L2 expression.

Table S2. Correlation of CD155, CD96 and TIGIT expression in tumor tissues. P value is calculated by Spearman's rho correlation. * The correlation is significant at the 0.01 level (2-tailed). ϱ= Correlation Coefficient. n= number of observations correlated.

| | correlation | CD155 | CD96 | TIGIT |
|---|---|---|---|---|
| **All** | | | | |
| CD155 | ϱ<br>p value<br>n | 1.000<br><br>139 | | |
| CD96 | ϱ<br>p value<br>n | **0.39***<br>**0.001**<br>139 | 1.000<br><br>139 | |
| TIGIT | ϱ<br>p value<br>n | 0.04<br>0.65<br>139 | **0.24***<br>**0.005**<br>139 | 1.000<br><br>139 |
| NOM | | | | |
| CD155 | ϱ<br>p value<br>n | 1.000<br><br>41 | | |
| CD96 | ϱ<br>p value<br>n | **0.653***<br>**< 0.001**<br>98 | 1.000<br><br>41 | |
| TIGIT | ϱ<br>p value<br>n | -0.084<br>0.601<br>41 | -0.265<br>0.094<br>41 | 1.000<br><br>41 |
| OSCC | | | | |
| CD155 | ϱ<br>p value<br>n | 1.000<br><br>98 | | |
| CD96 | ϱ<br>p value<br>n | **0.238**<br>**0.018**<br>98 | 1.000<br><br>98 | |
| TIGIT | ϱ<br>p value<br>n | 0.089<br>0.382<br>98 | **0.430**<br>**<0.001**<br>98 | 1.000<br><br>98 |

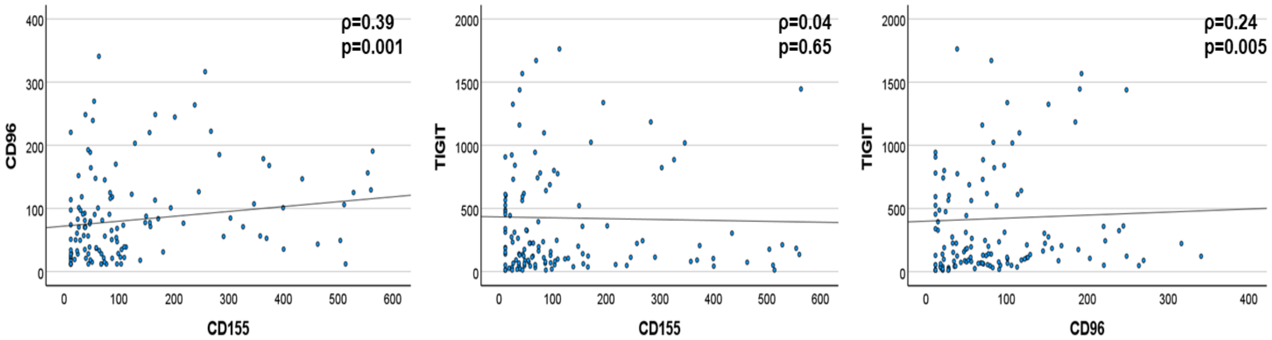


Figure S2. Correlation between the inhibitory ligand CD155 and its receptors CD96 and TIGT. Positive correlation between CD155 and CD96 expression could be shown. No correlation of CD155 expression and TIGIT could be proven. A positive correlation between CD96 and TIGIT expression could be determined.

Table S3. Correlation of CD115, CD163 and CD68. A) All samples were used. B) OSCC group. C) NOM group. P value is calculated by Spearman's rho correlation. * The correlation is significant at the 0.01 level (2-tailed). ϱ= Correlation Coefficient. n= number of observations correlated.

| | correlation | CD115 | CD163 | CD68 |
|---|---|---|---|---|
| **A) All** | | | | |
| CD115 | ϱ<br>p value<br>n | 1.000<br><br>139 | | |
| CD163 | ϱ<br>p value<br>n | 0.629*<br><0.001<br>139 | 1.000<br><br>139 | |
| CD68 | ϱ<br>p value<br>n | 0.589*<br><0.001<br>139 | 0.704*<br><0.001<br>139 | 1.000<br><br>139 |
| **B) OSCC** | | | | |
| CD115 | ϱ<br>p value<br>n | 1.000<br><br>98 | | |
| CD163 | ϱ<br>p value<br>n | 0.552<br><0.001<br>98 | 1.000<br><br>140 | |
| CD68 | ϱ<br>p value<br>n | 0.594<br><0.001<br>98 | 0.773<br><0.001<br>98 | 1.000<br><br>98 |
| **C) NOM** | | | | |
| CD115 | ϱ<br>p value<br>n | 1.000<br><br>41 | | |
| CD163 | ϱ<br>p value<br>n | 0.700<br><0.001<br>41 | 1.000<br><br>41 | |
| CD68 | ϱ<br>p value<br>n | 0.211<br><0.001<br>41 | 0.404<br>0.009<br>41 | 1.000<br><br>41 |

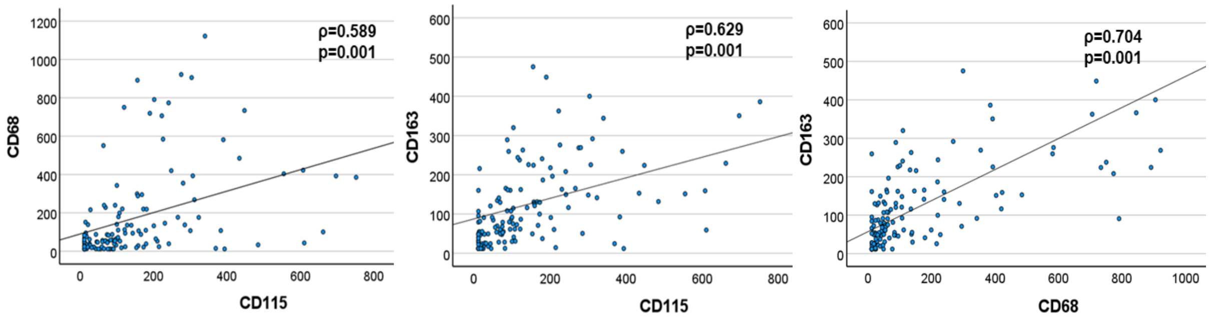


Figure S3. Positive significant correlations between CD115 expression and CD68 and CD163 (n=139). Expression of CD68 and CD163 was correlated strongly.

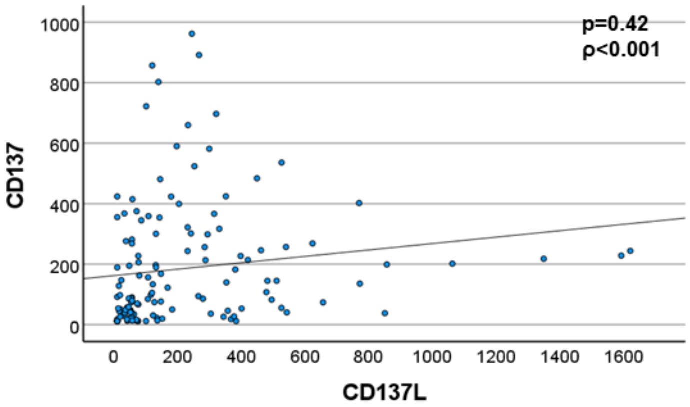


Figure S4. Expression of CD137 and of its ligand CD137L was strongly correlated.
